# Advancing bat monitoring: Assessing the impact of unmanned aerial systems on bat activity

**DOI:** 10.1371/journal.pone.0314679

**Published:** 2025-01-22

**Authors:** Marc Roswag, Anna Roswag, Matthias Sebastian Roswag, Joanna Fietz, Tessa Touridocht Taefi

**Affiliations:** 1 Department of Competence Center for Renewable Energies and Energy Efficiency, Hamburg University of Applied Sciences, Hamburg, Germany; 2 Department of Zoology, University of Hohenheim, Stuttgart, Germany; 3 Vespertilio–Faunistische Untersuchungen, Filderstadt, Germany; King Fahd University of Petroleum & Minerals, SAUDI ARABIA

## Abstract

With the increasing height and rotor diameter of wind turbines, bat activity monitoring within the risk area becomes more challenging. This study investigates the impact of Unmanned Aerial Systems (UAS) on bat activity and explores acoustic bat detection via UAS as a new data collection method in the vicinity of wind turbines. We tested two types of UAS, a multicopter and a Lighter Than Air (LTA) UAS, to understand how they may affect acoustically recorded and analyzed bat activity level for three echolocation groups: Pipistrelloid, Myotini, and Nyctaloid. We hypothesized (i) that the LTA UAS will not affect bat activity levels while a multicopter, due to higher noise emission, might have a negative impact. Our results support this hypothesis, because multicopter flights have a highly significant negative impact on bat activity levels with a medium effect size, particularly for the Myotini (*P* < 0.001, *d*_*m*_ = 0.54) and Nyctaloid group (*P* < 0.001, *d*_*n*_ = 0.55) and a small effect size for the Pipistrelloid group (*P* < 0.001, *d*_*p*_ = 0.36). In contrast, the LTA UAS had no significant effect on bat activity for each echolocation group (*P* > 0.05 for each group), suggesting its suitability for non-intrusive acoustic monitoring. Furthermore, we hypothesized (ii) that larger UAS propellers prevent the deterrent effect on bats. However, despite the use of larger propellers for the multicopter UAS compared to previous studies, we observed a deterrence effect for all echolocation groups. Additionally, we hypothesized that (iii) any initial deterrence or attraction effect might decrease over time. Our results did not support this hypothesis because we did not observe any habituation of bats to UAS within the 15-minute flight period. Our study highlights the potential of UAS for bat monitoring but underscores the critical importance of selecting appropriate UAS types and operating noise levels for successful surveillance efforts.

## Introduction

The increased use of renewable energy, particularly the expansion of wind energy, has become increasingly prominent during recent years, reflecting a global shift towards more sustainable power sources [1–4]. However, the increased numbers of operating wind turbines often conflicts with wildlife conservation [3–5]. Wind energy facilities in Germany alone cause the death of over 240,000 bats per year [6], significantly impacting bat (Microchiroptera) populations [7]. In order to establish mitigation strategies for bats, data on bat species diversity and bat activity levels close to wind turbines are collected [8]. Due to the fact that European bats are insectivorous and hunt using echolocation calls at ultrasonic frequencies, the most effective way to gather data on their activity is through acoustical recording of their echolocation calls [2]. In the context of wind energy projects acoustic bat surveys can be carried out (i) prior to the installation of a wind turbine with transect or permanent static surveys, and (ii) during the operation of wind turbines by nacelle monitoring. Transect surveys involve an expert walking on the ground with an acoustic bat detector to record bats within the area of a planned wind turbine. During permanent static surveys, several automatic bat recording devices are placed 2 meters above the ground nearby the planned wind turbine recording bat calls during an entire bat activity period (April to October) [8]. Both methods aim to collect data on bat activity to make predictions on the potential threat caused by the wind turbine. Nacelle monitoring involves placing a stationary acoustic detector on the nacelle of a wind turbine pointing toward the ground to record bat activity, verifying that the mortality risk for bats is not significantly increased during wind turbine operation. The acoustic detection of bats is severely limited by the high frequency (ultrasonic) range of their species-specific echolocation calls [2]. Shorter wavelength frequencies have a higher likelihood of interacting with particles and molecules in the atmosphere, leading to energy loss and a decrease in intensity as distance increases [9]. Therefore, during transect and permanent static surveys, an expert on the ground cannot acoustically detect bats in the risk area of wind turbine rotors. The risk area of a current average wind turbine is located between 60 meters and extends to at least 180 meters above the ground [10]. With the development of taller wind turbines, this average risk area will expand and shift upwards [11]. As with the height of wind turbines, the rotor sizes usually also increase [12,13]. Unfortunately, while the risk area per turbine increases, the detection range of the nacelle monitoring remains the same, leading to a lower cover of the total risk area, depending on the frequency range of the bat calls. Therefore, nacelle monitoring covers only 4% of the risk area of a wind turbine with 60 m blade length for bats emitting calls at 40 kHz, whereas 23% of the risk area is covered for bats emitting calls at 20 kHz [2]. The use of thermal imaging and radar techniques for the identification of flying bats is not a viable option, as neither can reliably identify the species nor recognize the object as a bat [2]. In order to overcome these limitations, these techniques are already being combined with acoustic detection, which enables species identification in a limited area [14]. However, ground-based detection sensors are currently not a viable option for effectively detecting bats in the risk area of wind turbines. Thus, the development of new methods to monitor bats at higher altitudes is needed [2].

One potential method to monitor bats in high altitudes is the detection of bat calls by using UAS [15,16]. UAS are increasingly applied in wildlife monitoring of several species like seabirds [17]. For the monitoring of wildlife in large or hard-to-reach areas, UAS are most commonly equipped with visual sensors [17–20]. However, this type of visual recording technology is not feasible for small nocturnal mammals. However, the acoustic recording of bat calls using UAS seems to be a promising monitoring method but poses technical and biological challenges.

### Technical challenges

#### Acoustic bat recording with UAS

During the past years, several technical challenges associated with the use of UAS for bat surveys have been pointed out. August & Moore [21] tested and compared the use of fixed-wing aircraft and multicopter UAS for acoustic bat detection. This comparison suggested that a multicopter produces lower noise emissions than a fixed-wing aircraft, and additionally, has the advantage of being able to record stationary data while hovering.

Multicopters with four propellers, also known as quadcopters, have been tested in further studies. These include DJI Spreading Wings S900 (3.3 kg and 15-inch propeller) [22], DJI Phantom 3 Pro (1.3 kg and 9-inch propeller) [15], DJI Phantom 4 (1.3 kg, 9.4-inch propeller) [23–25], DJI Mavic 2 Pro (907 g, 8.7-inch propeller) and DJI Mavic Mini (249 g, 4.7-inch propeller) [24] and DJI M210 V2 (4.69kg and 17-inch propellor) [16]. These multicopters emitted noise mainly below the 25–30 kHz frequency range [15,21,23]. A number of studies have sought to develop acoustic deterrent devices with the objective of reducing the activity of bats in areas where they are threatened. Gilmour et al. [26] demonstrated that the activity of Myotis and Pipistrelloid species was reduced by approximately 30%, while that of Nyctaloid species was reduced by approximately 70%. The DJI Phantom 4 was also used by Werber et al. [25] to induce deterrence effects by adding additional sources of interference such as loudspeakers (15–80 kHz) and flashing cold light. It was shown that bats below the deterrent device were frightened and increased their flight altitude, resulting in a significant increase in activity above the multicopter. Michez et al. [15] and Jespersen et al. [16] showed that the DJI Phantom 4 and the DJ M210 V2 emitted noise also in the 40 kHz frequency band and identified the electronic speed controllers as a potential source. They recommend testing an LTA UAS, such as a blimp, as a carrier for a bat detector [15,16]. An LTA UAS requires less propeller thrust, due to its helium buoyancy, and should be quieter than the quadcopters tested in previous studies. A multicopter with six propellers, that might produce different noise emissions [27,28], has not been tested yet.

To reduce the noise emitted by the UAS, disturbing the recording of bat calls, some studies tested the application of damping material between the UAS and the bat detector. Ednie et al. [23] and Jespersen et al. [16] used an acoustic foam to reduce the noise on the recording. Fu et al. [22] mounted a styrofoam insulating ball with a diameter of 15.24 cm at a height of 50 cm above the UAS. This measure reduced the noise of the UAS by up to 11 dB [22]. However, it should be noted that applying damping material also shields bat calls and reduces their sound level on the recordings. Thus, a second option to increase the signal-to-noise ratio might be to change the position of the bat detector.

There are three possible positions for attaching bat detectors to a multicopter: above, below, and lateral. However, lateral attachment is not feasible due to the highest noise generation occurring at a 90° azimuth direction (sideways) by the multicopter [29]. Furthermore, lateral attachment causes a significant shift in the center of gravity, which adversely affects flight characteristics [30]. Fu et al. [22] positioned the bat detector 50 cm above the UAS, which requires a rod due to working against gravity. This placement increases the weight of the UAS and the noise it produces, as the noise depends on the revolutions per minute of the propeller and the weight of the UAS, respectively. Furthermore, a stationary rod causes the payload’s weight to shift away from the multicopter center of gravity during maneuvers. This negatively impacts the multicopter’s flight characteristics, as tilting occurs during maneuvers [30]. To reduce the noise of a multicopter, it is necessary to position the bat detector at a certain distance from the rotors. The optimal placement is below the multicopter, where a string can be used for attachment, as opposed to the more complex and unstable installation of a pole above the multicopter [31].

#### Acoustic bat identification with superimposed UAS noise

Identifying bat calls when superimposed by UAS noise is technically challenging. Previous studies mainly identified bat calls by manual analysis of spectrograms [15,22,23]. Kuhlmann et al. [24] only used the automatic identification of bat calls by the Kaleidoscope software (Wildlife Acoustics 2023) and verified the automatically detected bat calls manually. Automatic analysis of bat calls by software has the disadvantage that calls are not reliably identified in the presence of noise, because automatic analysis focuses on the loudest signal (Volker Runkel, pers. communication). The multicopter tends to emit a louder signal than the recorded bat calls, which often leads to errors in identification, especially when using automatic software identification [31]. The use of Artificial Intelligence (AI)-based analysis could provide potential benefits in the future [31,32]. To date, previous research has shown that manual analysis is the most effective and reliable approach for obtaining accurate results in this scenario.

### Biological challenges of bat deterrence by UAS

Potential change in behavior of bats in the vicinity of an operating UAS poses a biological challenge for monitoring. The literature indicates that the response of bats to sounds in the ultrasonic range is species-specific [3,23–25,33–35]. To deter bats in the risk area of a wind turbine, Arnett et al. [34] conducted a study in which an ultrasound emitting device was placed on a wind turbine. The acoustic noise produced by the device ranged from 20 to 100 kHz and resulted in a significant decrease in bat activity. Since ultrasonic signals have limited range, Werber et al. [25] used a mobile UAS equipped with a deterrent device to deter bats to cover a larger area. The authors’ device emitted acoustic and visual pulsating signals specifically designed to deter bats. Their results indicated that the combination of audio and visual disturbance signals reduced the bat activity levels.

The impact of visual disturbance signals on bats has been explored in several studies [24,36–38]. These studies indicate that bats respond differently to various light sources according to their species. Kuhlmann et al. [24] specifically examined the behavior of bats in the presence of UAS signal lights. The study indicated that there was no statistically significant difference in bat passes between the use of UAS light and the control group without light. Overall, it appears that visual sources (position lights) of UAS have minimal or negligible impact on the behavior of bats.

Acoustic signals therefore play the most important role in the use of UAS for bat detection in terms of deterrence. In his study, Jesperson et al. [16] demonstrated that all the species under investigation exhibited a proximity of within one meter of the recording devices, which were situated at a distance of 30 m below the DJI M210 V2 with four 17-inch propeller. To our knowledge, only two studies have investigated the deterrent effect systematically on bats in the presence of UAS [23,24]. Both studies tested quadcopters of different sizes (DJI Mavic Mini: 249 g, DJI Mavic 2 Pro: 907 g, DJI Phantom 4: 1,380 g), each equipped with four propellers (DJI Mavic Mini: 4.7”, DJI Mavic 2 Pro: 8.7”, DJI Phantom 4: 9.4”). During the records by Ednie et al. [23] and Kuhlmann et al. [24] the multicopter flew at altitudes of 5–10 m [23] or 15 m [24] above the ground-based bat detector. Kuhlmann et al. [24] showed a significant decrease in the number of recorded and identified bat calls during UAS flights (DJI Mavic 2 Pro and DJI Phantom 4) compared to controls. However, they demonstrated that the operation of quadcopters has a significant impact on American bat species. To date, only one exceptionally small and lightweight UAS (DJI Mavic Mini– 249 g) has been identified that exhibits no discernible effect on the activity level of American bat species. It should be noted that the minimal weight of this UAS presents a challenge, particularly when equipping it with suitable detection sensors and when flying at elevated altitudes, where stronger winds, such as those that occur in the vicinity of wind turbines, can significantly compromise flight stability. In summary, the literature shows that monitoring bats is hindered by the noise emitted by multicopters. Kuhlmann et al. [24] suggest that smaller and quieter UAS do not deter bats, unlike larger and louder UASs. Further research is required to determine how bat activity levels are affected by the noise emitted by the UAS used for monitoring purposes. Moreover, it is currently unknown if the deterrence effect varies with distance from the UAS.

When examining an established deterrence effect, assessing the occurrence of a habituation effect over time can be advantageous. Kuhlmann et al. [24] conducted a study on this effect, which indicated that bats do not habituate to the presence of UAS during the flight time. However, this was only tested for a recording interval of 5-minutes and it remains to be tested whether a habituation effect could occur over longer flight durations. This study contributes to the existing body of research by examining the habituation effect over a flight duration of 15 minutes.

This study builds upon existing research on the use of UAS as a detection method for bats in inaccessible areas by examining novel UAS types, including hexacopters and LTA UAS. While only quadcopters have been tested so far, a hexacopter with 17-inch propellers is being used to shift the frequency band of the generated noise to lower ranges, thereby reducing the impact on the bats’ hunting echolocation calls. Preliminary results with four propellers have shown promising results [16], although systematic testing with this configuration has not yet been conducted, nor have tests with six propellers. This approach could result in the development of a UAS that is capable of withstanding stronger winds while also minimizing the impact on bats. The LTA UAS, which enables particularly quiet flight due to helium buoyancy, offers bat-friendly operation despite its size, which challenges the assumption proposed by Kuhlmann et al. [24] that smaller UAS are better suited for detection.

### Research questions

This study aims to answer the research question: 1) How do two different types of Unmanned Aerial Systems (UAS) (lighter than air (LTA)) UAS and a hexacopter with larger propellers) affect acoustically recorded activity levels of bats, in terms of attraction or deterrence? If there is an effect of the UAS on the bat activity 2) to what extent does the recorded acoustic bat activity change during a 15-minute UAS flight concerning a habituation effect?

We assumed that the LTA UAS has lower noise emissions than a multicopter due to its helium buoyancy and the associated lower take-off weight. Thus, we hypothesized (i) that the LTA UAS has no impact on bat activity levels. In addition, a multicopter equipped with larger propellers should shift its noise emission to a lower frequency range due to the lower number of rotations for the same thrust [39]. McKay et al. [39] showed in their study, that a multicopter with larger propeller configuration is generally quieter than the smaller ones. Therefore, we hypothesized that (ii) six larger propellers prevent the deterrent effect on bats. This is because the noise of the multicopter is shifted to a lower frequency range, which does not interfere with the echolocation calls of hunting bats. As a result, bats will not be disturbed and more bat calls should be identified, as their frequencies do not superimpose. Additionally, we hypothesize that (iii) any initial deterrent or attraction effect on bats during UAS flights decreases over time, resulting in a measurable return to baseline activity levels in a time frame of 15 minutes.

## Materials and methods

### Approval

The study was approved by Ethics Committee of the Hamburg University of Applied Sciences prior to the start. Each flight complied with all applicable drone flight laws (Commission implementing regulation (EU) 2019/947 and 2020/746 on the rules and procedures for the operation of unmanned aircraft and German air traffic regulations) and was manually controlled by a pilot with a flight license (e-ID = DEU48hakpl6va5su). All three study sites were public and unprotected areas for which the survey was agreed without the need of any permits by the respective town (City of Nürtingen, municipalities of Neckartenzlingen and Neckartailfingen). Furthermore, we considered the national animal and nature protection law and involved the relevant nature conservation authority, in this case the Lower Nature Conservation Authority of district Esslingen, which gave its unreserved approval.

### Selection of our study sites

To determine appropriate study locations, we investigated bat fauna over a period of one week during May 2022 using a stationary bat detector (Song Meter SM4BAT FS, Wildlife Acoustic) in 2 m height at six different study sites in close proximity to Stuttgart in the state of Baden-Württemberg, Germany. This setting is used in traditional bat activity surveys. Given the difficulty of flying UAS in forests, we prioritized areas at watersheds and at the forest edges. The findings of this survey led to our three study sites located at three water bodies, where almost all German-native bat species were present: Whiskered Bats (*Myotis mystacinus*), Natterer´s Bat (*Myotis nattereri*), Serotine Bat (*Eptesicus serotinus*), Common Noctule bat (*Nyctalus noctula*), Leisler’s Bat (*Nyctalus leisleri*), Long-Eared Bats (*Plecotus auritus*), Soprano Pipistrelle (*Pipistrellus pygmaeus*), Nathusius’ Pipistrelle bat (*Pipistrellus nathusii*), Kuhl’s Pipistrelle (*Pipistrellus kuhlii*), Daubenton’s Bat (*Myotis daubentonii*), and Common Pipistrelle (*Pipistrellus pipistrellus*). Two study sites are located at lakes (Aileswasensee at 48°36’N 9°15’E and Beutwangsee at 48°36’N 9°18’E) and one at a slowly flowing part of the river Neckar (48°35’N 9°13’E).

### UAS selection and used materials

We used two types of UAS that generate minimal acoustic emissions in frequency ranges above 20 kHz to reduce interference with the echolocation calls of bats and thereby reducing any deterrent effects. Accordingly, we used a multicopter (ConVecDro—Third Element Aviation GmbH) with six propellers, larger than those used in previous studies that systematically tested deterrence or attraction effects [23,24]. Based on the recommendations of Michez et al. [15] we used additionally a LTA UAS (Zero+–Hybrid Airplane Technology GmbH) that relies on helium gas for buoyancy to reduce the take-off weight and generates significantly lower levels of noise emission than multicopter systems (Table 1).

**Table 1:** Technical properties of tested UASs.

	ConVecDro Third Element Aviation	Zero + Hybrid Airplane Technology
** **Take-off weight** **	5 kg	0.1 kg
**Dimension** **(incl. propeller)**	143 x 143 x 53 cm	364 x 260 x 100 cm
** **Propeller (diameter)** **	17 “	7 “
** **Max. flight duration** **	30 min	120 min
**Noise / SPL** **during hovering**	79 dB	62 dB
** **Wind resistance** **	15 m/s	2 m/s

The LTA UAS is equipped with a 12-LED spotlight inside its hull to illuminate the UAS. This results in a diffused and indirect light. Additionally, in the hull near the propellers of the LTA UAS, two position lights are located, with green on the right side and blue on the left side. The lighting system for the multicopter includes a conspicuous light that conforms to the EN4709-004 standard and is equipped with green and red position lights that comply with the German aviation standard.

### Data collection

**Fig 1:**
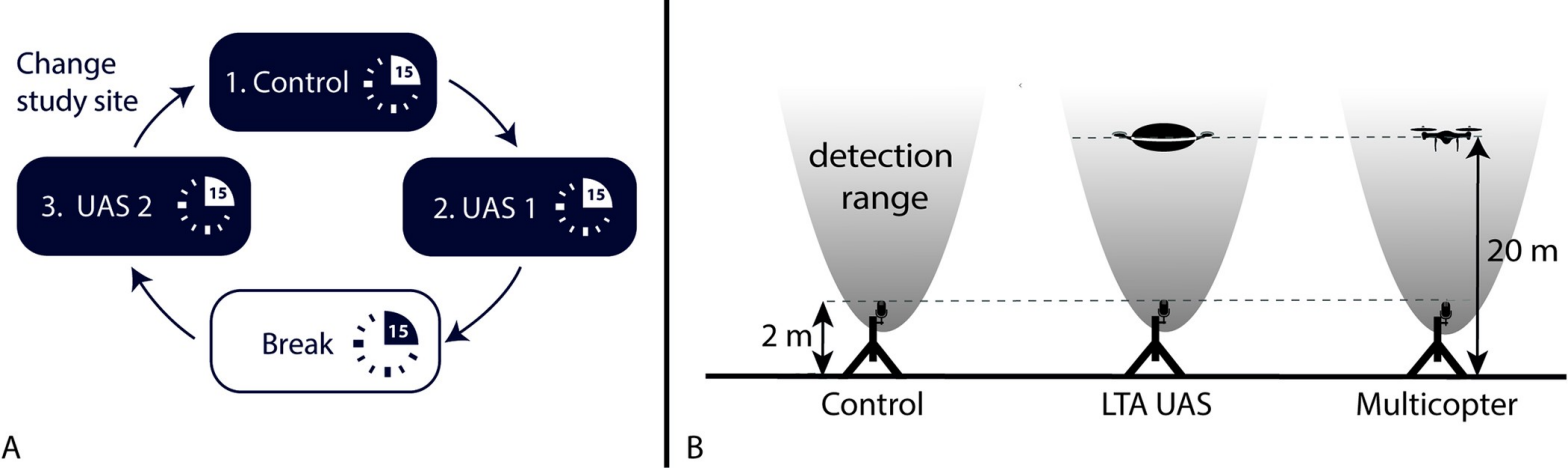
Illustration of the experimental design. (A) chronological sequence of recording phases per night (B) Test setup for each recording phase.

From June to the beginning of October 2022, we conducted recordings starting 30 minutes after sunset for a maximum of 4 hours. During each recording night, we performed a complete test setup consisting of phase 1 as negative control (15-minute recording without any UAS), phase 2 recording during flight of UAS 1, a 15-minute break, and a phase 3 recording during flight of UAS 2 (Fig 1A). To prevent potential effects of the UAS type on the bat activity level, we alternated the order between the LTA UAS and the multicopter each night. For each test setup, we measured the ambient temperature with a Multi-Function-Environment Meter (PeakTech 5035) from the ground and started the test only if it was above 10°C. Each recording lasted 15 minutes, resulting in a total test setup duration of 60 minutes before changing the study site. We choose a recording time of 15 minutes to achieve a representative data set and to examine a potential habituation effect for a longer period of time, in contrast to previous studies by Ednie et al. [23] and Kuhlmann et al. [24] who recorded for 5 minutes.

During all tests we used an automatic bat detector (Song Meter SM4BAT FS, Wildlife Acoustics, Inc.; sample rate: 500 kHz; gain: 12 dB; minimal duration: 1.5 ms; trigger window: 3 s; trigger level: 12 dB). The bat detector was placed two meters above the ground and was oriented vertically upwards to ensure optimal coverage of bats (Fig 1B).

The UAS started 10 m beside the bat detector and climbed to an altitude of 20m above ground level before navigating to the final recording point directly above the bat detector. The UAS hovered stationary directly above the bat detector during the recordings. However, the LTA UAS due to its susceptibility to winds, incurred a position variation of approximately ±1 m during hovering.

As the bat detector was placed 2 m above the ground, the distance between the bat detector and the UAS was 18 m (Fig 1B). At a distance of 18 m, all bats expected to be present in the region can be detected with echolocation calls with a peak frequency of up to 45 kHz around the UAS. This detection range is based on a study in which, for example, Common Pipistrelles (*Pipistrellus pipistrellus*) with a peak frequency of approximately 45 kHz have a median recording distance of 16–19 m, while Common Noctules (*Nyctalus noctula*) with a peak frequency of 20 kHz have a median recording distance of 39–42 m [2]. This setup therefore provides the maximum distance between the UAS and the bat detector to reduce noise from the UAS in the recording.

### Preparation of data for statistical analysis

We used the Kaleidoscope Pro software (Version 5.4.6 Wildlife Acoustics, Inc.) to split recordings into one-second files. Each file was then manually analyzed by clustering bat calls in three echolocation groups (Pipistrelloid, Myotini, Nyctaloid; Table 2) regarding their call characteristics.

**Table 2:** Categorization of bat species by echolocation group.

Echolocation group	Bat species
** **Pipistrelloid** **	*Pipistrellus pygmaeus* *Pipistrellus nathusii* *Pipistrellus kuhlii* *Pipistrellus pipistrellus*
** **Myotini** **	*Myotis mystacinus* *Myotis brandtii* *Myotis nattereri* *Myotis daubentonii* *Myotis bechsteinii* *Myotis myotis* *Plecotus auritus* *Plecotus austriacus*
** **Nyctaloid** **	*Nyctalus noctula* *Nyctalus leisleri* *Eptesicus serotinus* *Eptesicus nilssonii*

In the study conducted, Long-Eared Bats (*Plecotus auritus/austriacus*) was grouped with *Myotis* species due to their similar echolocation frequencies and foraging strategy of picking prey off surfaces. This classification is supported by the observation that the Long-Eared Bat uses echolocation frequencies within the range characteristic of *Myotis* species and shares a preference for a similar hunting strategy. Specifically, the gleaning behavior, observed in species such as the Greater Mouse-Eared Bat (*Myotis myotis*), Natterer’s Bat (*Myotis nattereri*), and the Bechstein’s Bat (*Myotis bechsteinii*), as well as *Plecotus* species such as the Brown Long-Eared Bat (*Plecotus auritus*), involves grabbing prey from the surface detected by their own rustling sounds. As a result, these species are highly sensitive to noise [33], which can significantly impair their ability to locate prey through rustling sounds, highlighting the importance of a quiet hunting environment for the effectiveness of their specialized foraging strategy.

Northern Bat (*Eptesicus nilssonii*) and Serotine Bat (*Eptesicus serotinus*) were categorized as ’Nyctaloid’ because both species predominantly hunt in open landscapes and exhibit similar low echolocation frequencies with a quasi-constant call sequence. These species are adapted to foraging in open habitats, where longer, low-frequency calls enable efficient prey detection and pursuit over greater distances. This categorization is based on the ecological and ethological characteristics that Northern Bat and Serotine Bat share with other species of the Nyctaloid group. The Nyctaloid group is characterized by their hunting strategy and acoustic signature.

One-second files were labeled as noise (no bat call identified) or as one or more of the three echolocation groups if calls from different echolocation groups were identified within a file. We did not use automatic labeling software, as it gives priority to the loudest signal (Volker Runkel, EcoObs GmbH, pers. comm.), which was often caused by UAS noise, resulting in incorrect identifications such as false-positive or false-negative. By comparing manual and automatic identification results, we found several challenges concerning bat call identification in the UAS noise. In the course of manual analysis of the project, approximately 65% of the recordings were labeled with at least one bat call, whereas only around 50% of the recordings could be identified as bat calls by the Kaleidoscope software. This consequently signifies that approximately 50% of the recordings remained unidentified. Of these, approximately 25% were labeled ’NoID’, indicating that although a bat was identified, its precise identification could not be confirmed. Such recordings would have to be subjected to manual examination in any case. Furthermore, it is not possible to ascertain whether multiple bat species were identified in a single recording, which could potentially lead to an inaccurate representation of the data. While there have been advancements in deep learning algorithms that have demonstrated superior outcomes [40,41], these were not incorporated into the present study. However, they could improve future studies, particularly in the analysis of recordings with high background noise, such as those produced by UAS.

Bat activity levels were determined by counting the number of one-second files that contained the respective echolocation group for each phase. Within the 15-minute recording interval, bat activity ranged from 0 to a maximum of 900 one-second files (corresponding to 900 seconds in 15 minutes). To determine the potential habituation effect, the activity level was calculated for each treatment on a one minute basis, resulting in a maximal activity of 60 one-second files.

**Fig 2:**
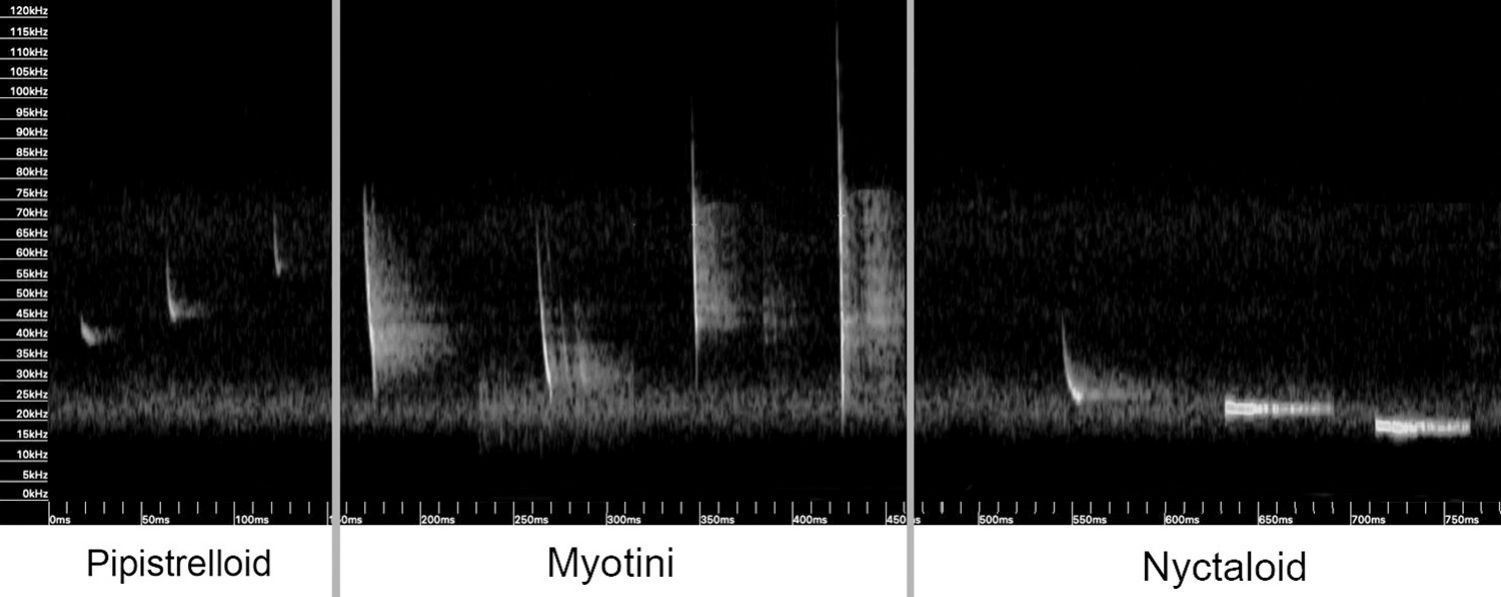
Sonagrams of echolocation calls categorized by Pipistrelloid, Myotini, and Nyctaloid bats.

The dataset included several variables that were critical to the subsequent analysis. It includes three study sites–Aileswasensee, Beutwangsee and Neckar as factors in the *Location* variable. The *Treatment* factor variable indicates whether a Multicopter or LTA UAS was used during the test, or whether no UAS was used as a control. The *JD* (Julian Day) variable represents the night on which a given treatment occurred, expressed as a numeric value converted to Julian date format. *Treatment_minute* denotes a fixed minute during a treatment. As the duration of a treatment is 15 minutes, this variable contains a number from 0 to 14. The *Species* variable contains one of the bat species (Fig 2). The *ID* variable serves as a unique identifier for each series of records and combines the values of *Location*, *Treatment* and *JD*. The Activity variable indicates the number of 1-second files for one echolocation per phase.

### Statistical analysis

The statistical software R (version 4.2.1) was used to conduct the analyses. To investigate potential deterrence or attraction effects, we employed a linear mixed effects model (LMER) using the lme4 package (version 1.1.31) [42]. Normality of the data was confirmed using the Shapiro-Wilk test, and homoscedasticity of residuals was assessed using the Breusch-Pagan test. If the Shapiro-Wilk test indicated a deviation from normal distribution assumption, we applied various data transformations, including square root (SQRT), logarithmic (Log), and Box-Cox transformations with lambda equal to 2. The transformation that yielded the best approximation to normality was selected for further analysis. If the requirements cannot be met, such as in cases where achieving a normal distribution is not possible even after transformations, a general linear mixed model (GLMM) with a Poisson distribution from the same lme4 package was used. These models aimed to examine the relationship between activity as the response variable per species (Nyctaloid, Myotini, Pipistrelloid) and treatment (control, multicopter or LTA UAS) as a fixed effect explanatory variable. We accounted for variability in activity between days and locations by treating Julian Day (JD, nightly date) and location (Aileswasensee, Beutwangsee and Neckar) as grouping random effects.

For the linear mixed effects model, general linear hypothesis tests were conducted to examine contrasts between acoustically detected bat activity during UAS flights and the control. The ’glht’ and the ’mcp’ function from the ’multcomp’ package (version 1.4.20) were used to calculate the Tukey contrast for the test setup.

If a significant difference was found, Cohen’s d was calculated to quantify the effect size (lsr package, version 0.5.2). This parameter provides a standardized measure of effect size, allowing us to assess the magnitude of the difference.

To assess the goodness of fit of each model, we calculate the coefficient of determination (R^2^) using the ’rsq’ function from the ’rsq’ package (version 2.5).

If a significant difference was observed between the control and UAS flight recordings, we aimed to confirm the presence of a habituation effect during the UAS flight by using a general linear mixed-effects regression (GLMER). In this model, we used activity per echolocation group as the response variable and the treatment minute (which denotes a fixed minute during a treatment ranging from 0 to 14) as the fixed effect explanatory variable. To account for variability in activity between different days and locations, we included JD and location as grouping random effects. We used a Poisson distribution for this model.

## Results

**Fig 3:**
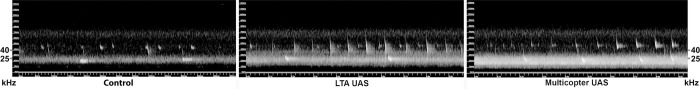
Sonagrams of bat calls recorded during different experimental phases. Each panel displays representative sonagrams for the echolocation groups Pipistrelloid, Myotini, and Nyctaloid.

We recorded a total of 107,356 one-second files. 35.4% (38,004) did not contain any bat calls and were therefore labelled as noise. Of the 69,352 files containing bat calls, 89.3% (61,915) were attributed to Pipistrelloid, 18.4% (12,743) to Myotini and approximately 3.9% (2,701) to Nyctaloid. It should be noted that a single file could contain calls of all three echolocation groups. However, bats emitting echolocation calls below 25 kHz, such as *N*. *noctula*, posed more identification challenges due to the noise produced, particularly by the multicopter (Fig 3). During the recordings, ambient temperature ranged between 11°C and 24°C, with an average of 16°C.

### Deterrence effect

**Fig 4:**
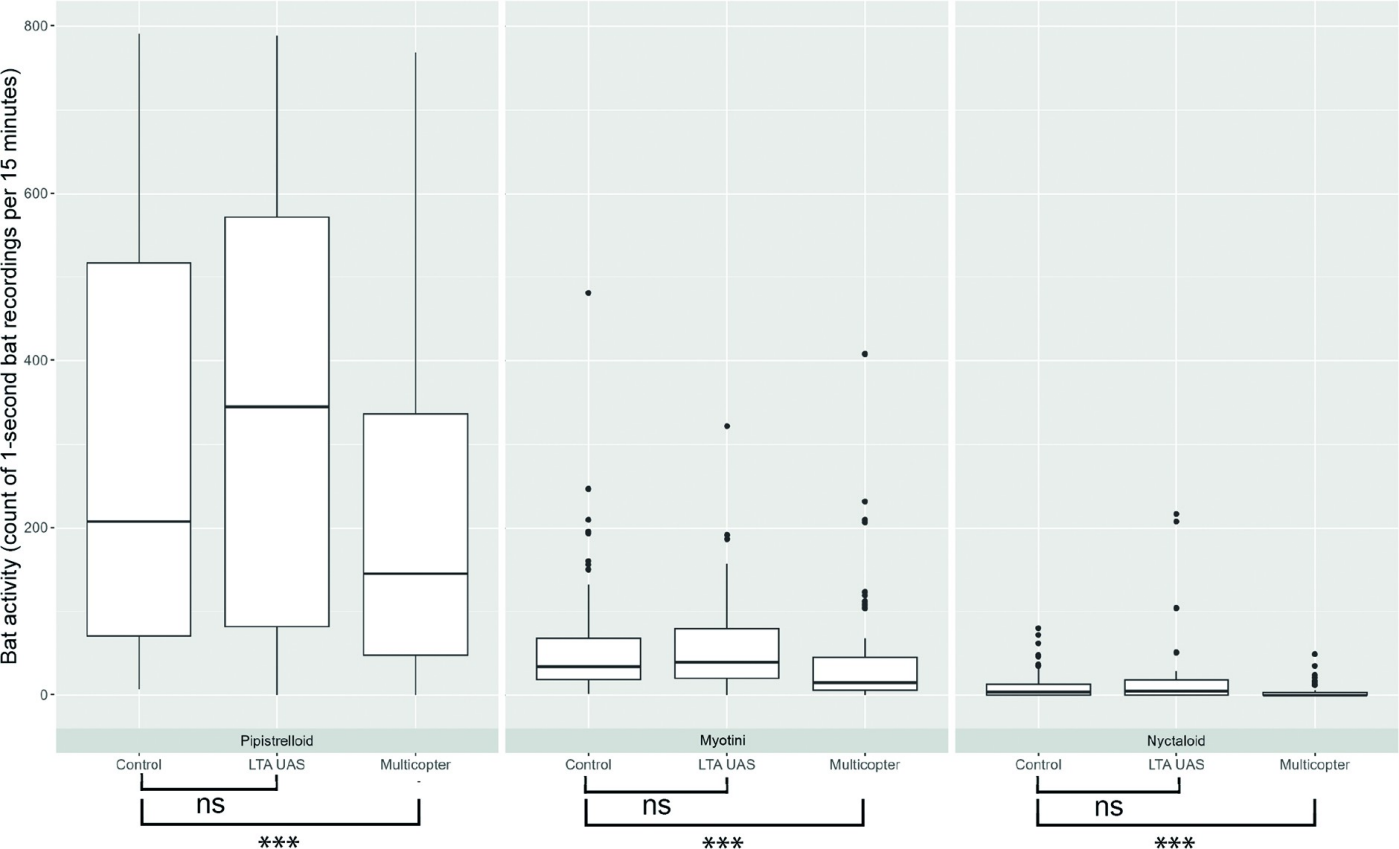
Bat activity levels (count of 1-second recordings per 15 minutes) for each echolocation group and treatment condition. Shown are median (line) with 25%-75% quartiles (box), smallest and largest non-outlier points (whiskers) and outlier (points). Significant differences are marked by stars (*** P < 0.001) and non-significant differences are marked by “ns”.

Statistical models did not reveal a significant difference in bat activity during flight with the LTA UAS compared to the control (for all comparisons *P* > 0.23). This finding applies to the analysis of all three echolocation groups n = 43 (LMER: Pipistrelloid, *P* = 0.81, *R^2^* = 0.87, Myotini, *P* = 0.23, *R^2^* = 0.86, Nyctaloid, *P* = 0.61, *R^2^* = 0.32). In contrast, a significant difference of bat activity was observed during flight with the multicopter n = 63 for all three bat species (LMER: Pipistrelloid *P* < 0.001, *R^2^* = 0.82, Myotini *P* < 0.001, *R^2^* = 0.72, GLMM: Nyctaloid *P* < 0.001, *R^2^* = 0.65). For all their echolocation groups significantly less bat activity was identified during the multicopter flight compared to the control (post-hoc Tukey for all comparisons: *P* < 0.001, Fig 4, S1 Table).

**Fig 5:**
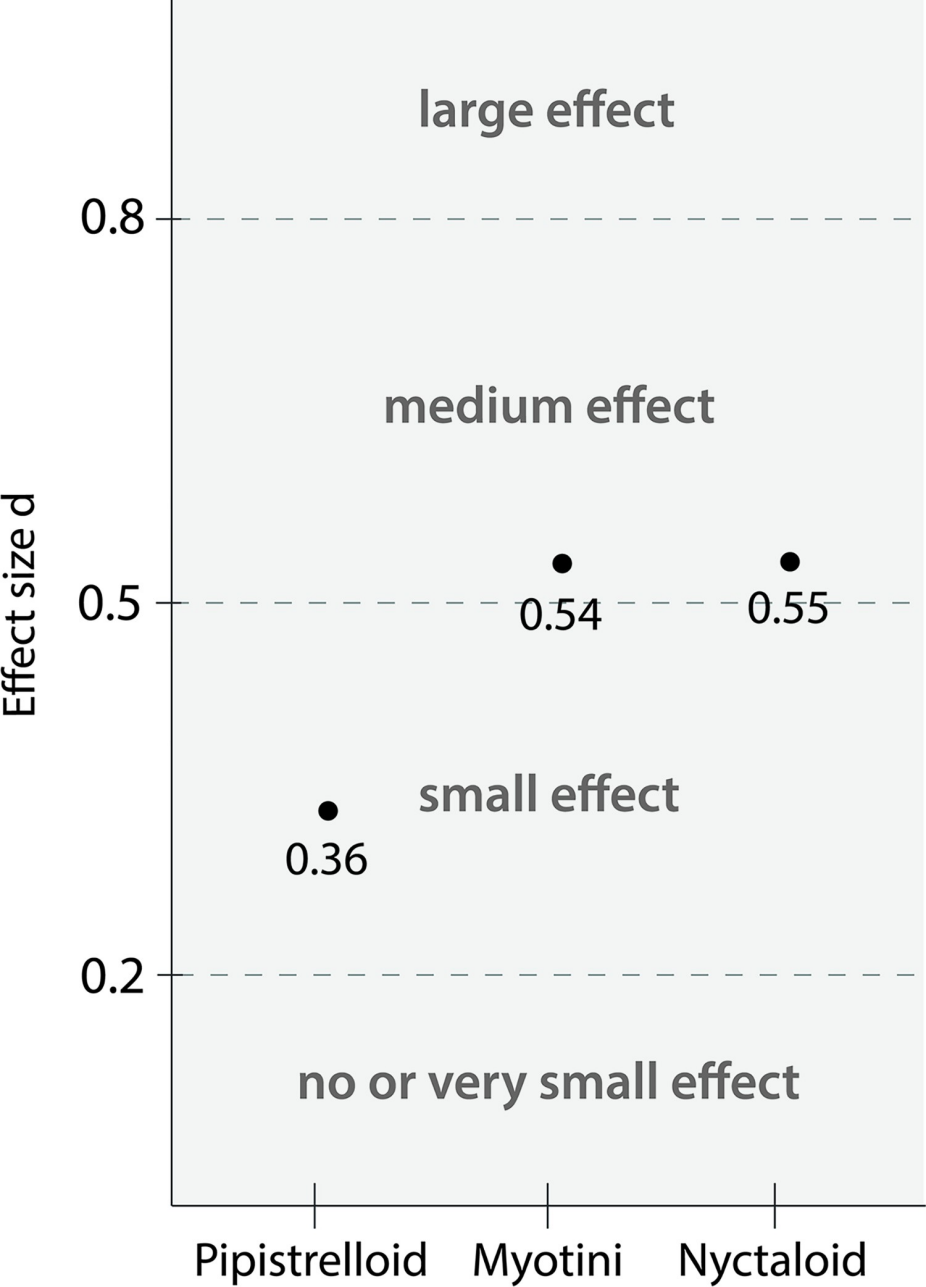
Cohen’s d effect sizes for the multicopter treatment across different echolocation groups.

The calculated effect sizes for models with a significant difference provided insight into the impact of the multicopter. According to Cohen’s thresholds for interpreting effect sizes, we observed that the presence of the multicopter had a small effect on Pipistrelloid species (*d*_*p*_ = 0.36, Fig 5). In contrast, the effect sizes for Myotini (*d*_*m*_ = 0.54, Fig 5) and Nyctaloid species were medium (*d*_*n*_ = 0.55, Fig 5).

### Habituation effect

The habituation effect analysis was only performed for the multicopter data, as the LTA UAS had no significant effect on bat activity. The analysis found a significant habituation effect for the Pipistrelloid group (*P* < 0.01, *R^2^* = 0.75, estimated slope < 0.01), but not for the Myotini group (*P* > 0.05, *R^2^* = 0.62, estimated slope < -0.01) or the Nyctaloid group (*P* > 0.05, *R^2^* = 0.17, estimated slope = 0.02, S2 Table).

**Fig 6:**
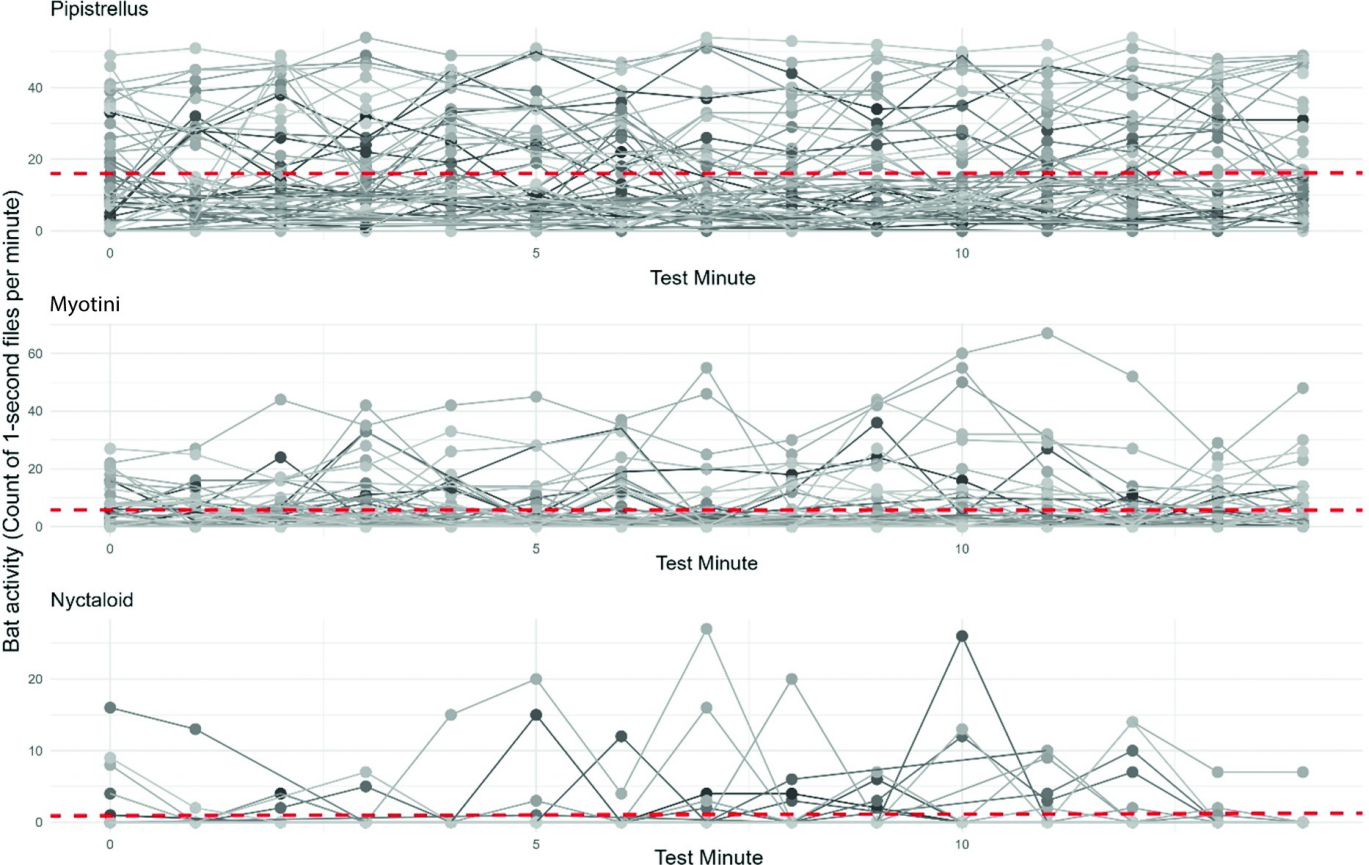
Bat activity changes (count of 1-second files per minute) over a 15-minute period during multicopter flight for different echolocation groups; Each grey line represents one 15-minute recording period. The red dashed line indicates the average bat activity at test minute 0 and progresses according to the estimated slope derived from the GLMER.

These results show that a habituation effect is not discernible in all three cases, based on the estimated slope in bat activity per minute (Fig 6).

## Discussion

In accordance with our hypothesis, the results showed that the two types of UAS have different effects on the acoustic recording of bat activity. Hypothesis (i) that the LTA UAS has no impact on bat activity levels can be accepted based on the study results, which showed no statistically significant effect of LTA UAS flights on bat activity compared to a control group without UAS. This observation applies to all echolocation groups studied. We assume that the main reason for this is the low and intermittent noise emission. Switching the propellers on and off to regulate altitude occasionally stops the noise of the rotating propellers, partly completely, so there is no continuous noise. In addition to the minimal noise pollution generated by the LTA UAS, the indirect lighting emitted by the subtly illuminated, non-glaring cover may also represent an attractive feature for the bats. It is therefore possible that this potential attraction effect could compensate for the noise pollution, and thus lead to a similar result to that observed in the negative control, which showed no significant differences from the treatment phase during the flight with the LTA UAS. The illumination of a multicopter was found to have no measurable effect on bat activity in a former study [24]. However, other studies have indicated that different light sources can elicit varying responses in bats, including both deterrence and attraction [3,37,38]. This suggests that the type of light source may play a role for bats, which should be investigated in future studies.

Hypothesis (ii) stated that the use of a hexacopter with six larger propellers, which shifts noise emissions into lower frequency ranges, prevents a deterrence effect compared to previous studies [16,23,24]. Despite the distance of 18 m between the UAS and the bat detector, our data showed that all echolocation groups studied significantly reduced their activity during the operation of the multicopter compared to the control group without UAS presence. The findings of this study indicate that while six larger propellers are capable of generating noise in lower frequency ranges, the weight of the UAS also exerts a significant influence on noise generation. A higher weight is often associated with a larger multicopter. In order to further optimize the acoustic detection of bats with hexacopter, the use of six larger propellers is recommended, but only if this does not significantly increase the weight of the hexacopter. Further tests are necessary to investigate how propellers of different sizes affect the deterrence of bats with the same weight and type of UAS [35]. However, the observed effect varied among different echolocation groups, suggesting a possible correlation between the peak frequencies of their echolocation calls and their different sensitivity to noise, which is also assumed by previous studies [23,24]. Our results showed that the hexacopter flight had only a small effect on the Pipistrelloid echolocation group, with their peak frequency averaging around 40 kHz. For the Myotini echolocation group, with their highly frequency-modulated range of 20–80 kHz due to frequency-modulated calls, as well as the Nyctaloid echolocation group, which produce lower-frequency calls (peak frequency <25 kHz), we observed a medium effect of the multicopter flight. The Myotis species with highly frequency-modulated echolocation calls, especially those with gleaner behavior, have a higher sensitivity to auditory disturbances [33]. Furthermore, the species belonging to the Plecotus species, which have been grouped together with the Myotis species in this study, exhibited a significant sensitivity to frequencies in the range of 7–20 kHz [43]. This sensitivity for noise and the frequency range superimposed by the noise generated by the multicopter and could explain the greater effect size in the significant reduction in identified calls of the Myotini echolocation group during mutlicopter flights. Lohith et al. [35] demonstrated in their laboratory-based measurements that gleaning bats can hear the noise of a UAS better than other species and are therefore more susceptible and affected. The greater effect size for the Nyctaloid echolocation group may be due to the fact that these echolocation groups were often unidentifiable in the multicopter noise due to their low frequency calls and should be interpreted cautiously. In addition to the disturbing noise, the downwash from the multicopter can affect the activity level of the bats. For practical use, it is generally recommended to place the microphone further below the UAS and in the opposite direction of the multicopter. This not only further reduces the noise generated by the multicopter, but also minimizes the influence of the downwash from the multicopter.

Our findings align with previous studies [23,24], indicating that bats emitting higher-frequency calls are less affected by a deterrent effect than bats with lower frequency calls. Interestingly, Ednie et al. [23] reported the highest impact on Myotis species (22% of compared control groups), whereas Kuhlmann et al. [24] suggested less impact. Regarding species with lower-frequency echolocation calls (Nyctaloid in our study), Kuhlmann’s et al. study [24] demonstrated a strong deterrent effect, whereas Ednie et al. [23] indicated less impact. We assume that the difference in effect sizes between Myotini and Nyctaloid species in these two studies is due to the varying distances between the UAS and bat detector. Ednie et al. [23] positioned the bat detector 5–10 m away from the UAS, while Kuhlmann et al. [24] chose a distance of 15 m. The shorter distance results in higher frequency recording of UAS noise, making acoustic identification more challenging, particularly by software. Moreover, Myotis species emit their echolocation calls at lower volumes, further complicating their identification amidst background noise [44]. The noise in the 40 kHz band, which was measured in previous studies [15,23] was supposed by the UASs electronic speed controllers, which was not visible in our recordings. This could also provide an explanation for the increased effect size observed in Myotis species, as reported by Ednie et al. [23]. Notably, both studies included American bat species with different frequency echolocation calls as the European bats in our study.

We hypothesized that the decrease in identified bats calls during multicopter flight is mainly caused by the noise emitted by the multicopter. Previous research has documented that this noise interferes with the bats’ hunting abilities and impairs their overall activity [33,43,45]. Consequently, researchers have explored the development of targeted deterrence methods for bats, such as airflow devices and similar technologies, to reduce their presence in sonicated areas [34,46,47]. The multicopter used in our study generated noise emissions detectable up to 20 kHz at a distance of 18 m. Beyond this distance, sound levels were only recorded below 20 kHz due to sound propagation in the atmosphere. Kuhlmann et al. [24] highlighted that bats closer to the UAS than the recording device may be exposed to higher frequencies than those recorded, potentially resulting in an increased effect size, particularly for Myotini the echolocation group. Furthermore, it is suspected that the decrease in activity among Nyctaloid echolocation groups during the multicopter flight is primarily caused by identification difficulties. Some calls may be lost in the background noise emitted by the multicopter, particularly if they are quieter due to their greater distance from the bat detector. This could result in a misinterpretation of the term ’deterrence’. It is possible that models trained in the field of deep learning may prove more effective than traditional methods in identifying bat calls, even when noise levels are high.

In the present study, the activity of bats in the presence of different, yet untested UAS types was investigated. To achieve this, we positioned the bat detector 18 m below the UAS and pointing upwards. As a result, the recording area includes the area with the highest UAS noise emission. As demonstrated by earlier research [24], acoustic emission might be the primary factor of the deterrent effect. Thus, ideally, the bat detector would be oriented away from the UAS in a real-life scenario. This could on the one hand improve the detection and analysis of bat calls due to less UAS-caused noise in the recordings and on the other hand the recording area would then cover less noise affected parts where bats would be less disturbed. Therefore, future research could focus on this idea.

Our initial hypothesis (iii) was that due to habituation bat activity levels would return to baseline during a 15 minute flight. In our case, this means that the bat activity levels would increase in the case of deterrence effects. Previous studies utilized a recording time of 5 minutes and did not find a significant habituation effect during this flight time. The present study builds upon existing research by examining the impact over an extended flight duration of up to 15 minutes. Our findings showed no effect in two echolocation groups and only a minor effect in the echolocation group Pipistrelloid. The statistical model for this echolocation group demonstrated a significant correlation between time per minute and bat activity, the calculated increase in one-second files per minute was only 0.02. From a biological perspective, this increase is insufficient to be considered a meaningful effect. Therefore, we conclude that no habituation effect was evident within this timeframe of 15 minutes, leading us to reject our hypothesis. Future studies could investigate whether a habituation effect could result from longer and continuous monitoring over several months with the recurring use of survey UAS.

The investigation showed that the LTA UAS is more sensitive to wind conditions than the multicopter UAS. Specifically, the flight stability of the LTA UAS is significantly compromised when wind speeds exceed 1 km/h. During our test runs, we had to interrupt the test three times due to lateral gusts of around 5 km/h, which caused significant drift in the LTA UAS. During daylight flight tests, updrafts were a more significant problem, the intensity of which we were unable to measure. This vulnerability is due to the aerodynamic configuration of the LTA UAS, which has a larger target area for thermal updrafts and crosswinds due to its exposed projection area. While the oval shape of the helium hull appears to be optimal for flight and offers superior protection from lateral winds, it is important to consider that this shape presents a considerable surface area for buoyancy forces. However, as these buoyancy forces occur less frequently at night, we conclude that the shape is suitable overall, particularly for this specific area of use. The small maneuvering propellers were insufficient to counteract the intensified wind forces under these conditions. The utilization of a LTA UAS is regarded as a promising avenue of research, given its suitability for recording bats. This is due to the extended flight times, quiet flight behavior and the potential for discreet lighting afforded by the system. However, the current limitations of this technology, namely its high susceptibility to wind, necessitate the implementation of optimization measures. Based on our research, we propose a reduction in the size of the helium envelope, which would serve to diminish the surface area exposed to the wind, even if this results in a reduction in lift force. Furthermore, the utilization of larger side propellers could enhance maneuverability while simultaneously minimizing noise pollution in the higher frequency ranges.

## Conclusion

In conclusion, this study examines the impact of two previously unexamined types of UAS on European bats using acoustic detection to improve bat activity monitoring within the risk area of wind turbines. Here, we tested a hexacopter with propellers that are nearly twice the size (17 inches) of the largest propellers (9.4 inches) previously tested in comparable studies on quadcopters. Furthermore, for the first time an LTA UAS (lighter-than-air unmanned aircraft) was examined for acoustic bat monitoring, which primarily gains lift through helium, thereby enabling longer flight times and quieter flight.

Our findings indicate that UAS with lower noise emissions are suitable for bat detection, regardless of their size. However, it is important to consider that practical implementation requires a UAS with stable flight characteristics. According to this study, LTA UAS may be a more appropriate choice for bat surveys in inaccessible locations, given its minimal disturbance effect. However, further optimization is necessary to address its high susceptibility to wind. The tested hexacopter showed high wind stability but affected bat activity negatively, probably due to higher noise emission. This might be reduced by using a lighter hexacopter with same propeller size. A potentially viable solution could be a UAS that is partially lifted by helium to minimize the take-off weight. Additionally, to reduce susceptibility to wind, the gas cell should remain compact. Larger and more powerful side propellers could help to improve maneuverability in strong winds. We could show that the larger LTA UAS did not affect bat activity.

## Supporting information

S1 Table Conclusion of statistical results for deterrence or attraction effect—LTA UAS and multicopter. (DOCX)

S2 Table Conclusion of statistical results for habituation effect—multicopter. (DOCX)
