# Supplementary material for: Advancing bat monitoring: Assessing the impact of unmanned aerial systems on bat activity
Source: PLoS One. 2025 Jan 22;20(1):e0314679. doi: 10.1371/journal.pone.0314679 (PMC11753712; doi:10.1371/journal.pone.0314679)
Supplement: S1 Table — (DOCX) [file pone.0314679.s001.docx]

| **UAS** | **Echolocation Group** | **Model** | **P-Value** | **R²** | **Effect Size d** |
| --- | --- | --- | --- | --- | --- |
| **LTA UAS**  **(Zero +)** | Pipistrelloid | LMER | 0.81 | 0.87 | No effect |
|  | Myotini | LMER | 0.23 | 0.86 | No effect |
|  | Nyctaloid | LMER | 0.61 | 0.32 | No effect |
| **Multicopter**  **(ConVecDro)** | Pipistrelloid | LMER | < 0.001 | 0.82 | 0.36 |
|  | Myotini | LMER | < 0.001 | 0.72 | 0.54 |
|  | Nyctaloid | GLMM | < 0.001 | 0.65 | 0.55 |
