# Supplementary material for: Advancing bat monitoring: Assessing the impact of unmanned aerial systems on bat activity
Source: PLoS One. 2025 Jan 22;20(1):e0314679. doi: 10.1371/journal.pone.0314679 (PMC11753712; doi:10.1371/journal.pone.0314679)
Supplement: S2 Table — (DOCX) [file pone.0314679.s002.docx]

| **UAS** | **Model** | **P-Value** | **R-Value** | **Estimated Slope** |
| --- | --- | --- | --- | --- |
| **Multicopter**  **(ConVecDro)** | Pipistrelloid | < 0.01 | 0.75 | < 0.01 |
|  | Myotini | > 0.05 | 0.62 | < -0.01 |
|  | Nyctaloid | > 0.05 | 0.17 | 0.02 |
